# The impact of vestibular function on cognitive–motor interference: a case–control study on dual-tasking in persons with bilateral vestibulopathy and normal hearing

**DOI:** 10.1038/s41598-023-40465-2

**Published:** 2023-08-23

**Authors:** Maya Danneels, Ruth Van Hecke, Laura Leyssens, Raymond van de Berg, Ingeborg Dhooge, Dirk Cambier, Stefan Delrue, Vincent Van Rompaey, Leen Maes

**Affiliations:** 1 Department of Rehabilitation Sciences, Ghent University, Corneel Heymanslaan 10, 9000 Ghent, Belgium; 2 Department of Otorhinolaryngology and Head and Neck Surgery, Maastricht University Medical Center, Maastricht, The Netherlands; 3 Faculty of Physics, Tomsk State Research University, Tomsk, Russia; 4 Department of Otorhinolaryngology, Ghent University Hospital, Ghent, Belgium; 5 Department of Head and Skin, Ghent University, Ghent, Belgium; 6 Department of Otorhinolaryngology and Head and Neck Surgery, Sint Lucas Hospital, Ghent, Belgium; 7 Department of Otorhinolaryngology and Head and Neck Surgery, Antwerp University Hospital, Edegem, Belgium; 8 Faculty of Medicine and Health Sciences, University of Antwerp, Antwerp, Belgium

**Keywords:** Auditory system, Cognitive neuroscience, Learning and memory, Oculomotor system, Sensorimotor processing, Medical research, Neurology

## Abstract

Bilateral vestibulopathy (BV) is a chronic vestibular disorder, characterized by bilaterally absent or significantly impaired vestibular function. Symptoms typically include, but are not limited to, unsteadiness and movement-induced blurred vision (oscillopsia). This prospective case–control study aimed to elucidate the impact of BV on cognitive and motor performance and on cognitive–motor interference. Cognitive and motor performance, as well as cognitive–motor interference were measured in persons with BV and normal hearing using the 2BALANCE dual-task protocol. The experimental group was matched to a healthy control group based on age, sex, and educational level. The 2BALANCE protocol comprises cognitive tests assessing visuospatial memory, mental rotation, visual and auditory response inhibition, visual and auditory working memory, and processing speed. The cognitive tests were performed in single-task condition (while seated), and in dual-task condition (during a static and a dynamic motor task). The static motor task consisted of balancing on a force platform with foam pad. The dynamic motor task consisted of walking at a self-selected speed. These motor tasks were also performed in single-task condition. A generalized estimating equations model was used to investigate group differences for all cognitive and motor outcome measures. The estimated marginal means, as well as the odds ratios (OR), and their 95% confidence intervals (CI) were calculated. For the backward digit recall test, a baseline measurement was performed and analyzed using a student-t test. A total of 22 patients with BV and normal hearing and 22 healthy control subjects were assessed [mean age (SD), BV = 53.66 (13.35) and HC = 53.21 (13.35), 68% male]. The BV group had poorer mental rotation skills in single-task condition, compared to the control group [odds ratio (OR) = 2.30, confidence interval (CI) = 1.12–4.73, P  =  0.024]. Similarly, auditory and visual working memory were also poorer in the BV group in single-task condition (P = 0.028 and P = 0.003, respectively). The BV group also performed poorer on the mental rotation task and the visual response inhibition task in dual-task condition (OR = 2.96, CI = 1.57–5.59, P  <  0.001 and OR = 1.08, CI = 1.01–1.16, P  =  0.032, respectively). Additionally, an interaction effect, indicating increased cognitive–motor interference in the BV group, was observed for mental rotation, response inhibition, and auditory working memory (P  =  0.003 to 0.028). All static motor outcome parameters indicated more postural sway in the BV group compared to the control group for all test conditions (P  <  0.001 to 0.026). No group differences were noted for the dynamic motor task. These findings suggest a link between vestibular function and cognitive performance, as well as a greater interference between cognitive and motor performance in BV, compared to healthy controls.

## Introduction

Bilateral vestibulopathy (BV) is a chronic vestibular disorder, characterized by bilaterally absent or significantly impaired vestibular function^1^. Symptoms typically include, but are not limited to, unsteadiness and movement-induced blurred vision (oscillopsia). This may result in postural imbalance while standing and walking, causing an increased fall risk compared to the healthy population, but also compared to persons with other vestibular dysfunctions^2,3^. Aside from these motor deficiencies, cognitive impairment has also been observed in BV. The visuospatial cognitive subdomain was identified as affected most frequently and was linked to a decrease in hippocampal volume, possibly resulting from a lack of bilateral vestibular input^4–6^. Additionally, deficits in other cognitive subdomains such as attention, memory, processing speed, and executive function have also been reported^7,8^.

The combined impact of BV on cognitive and motor functioning could potentially result in cognitive–motor interference. In predictable environments, postural control is fairly automatic for healthy persons. However, in dynamic situations and/or in conditions such as BV, this process can require more attentional resources^7^. According to Kahneman’s Capacity Model of Attention, this increased requirement for attentional resources to maintain postural control, may result in a decrease in cognitive reserve^9^. Consequently, this may interfere with a simultaneously performed cognitive task and may result in cognitive–motor interference.

Cognitive–motor interference is typically assessed using a dual-task design. Thus far, only two studies assessed cognitive–motor interference in persons with BV. First, Bessot et al. (2012) were unable to identify any motor or cognitive differences between the patient and control group in single-task setting, nor any cognitive differences in dual-task condition^10^. However, the BV group did have a slower gait speed on the motor task in dual-task condition. The used cognitive task consisted of counting backwards by two, which does not evaluate a specific cognitive domain. Consequently, this task may not have been sufficiently sensitive to identify cognitive deficiencies in single, nor dual-task settings. Second, Sprenger et al. (2017) studied postural stability in various conditions, such as while counting backward^11^. However, they did not study cognitive performance. Therefore, the 2BALANCE protocol, which assesses various cognitive and motor skills, was developed and verified for its test–retest reliability^12,13^. For this investigation, it was hypothesized that persons with BV perform worse on the cognitive and motor tasks in single-task condition, and that they might perform disproportionally worse on cognitive and motor dual-tasks; i.e. they might show greater cognitive–motor interference than healthy persons.

## Material and methods

### Study design

This case–control study took place at Ghent University. Patients were recruited via Ear-Nose-Throat specialists, affiliated at Ghent University Hospital, Antwerp University Hospital, Maastricht University Medical Center, and General Hospital Sint-Lucas Ghent. All participants were assessed during a three-hour visit, where cognitive–motor dual-tasks and audiovestibular tests were performed by a certified audiologist (first author). Testing only took place in the morning, aiming to limit loss of attention caused by fatigue. A learning effect was prevented at group level by developing nine different randomization sets. The instructions were presented in a visual and auditory manner to ensure correct understanding. Participants gave their written informed consent prior to participation in accordance with the Declaration of Helsinki. This study was approved by the ethics committee of Ghent University Hospital (B670201940465) and was registered in clinicaltrials.gov (NCT04126798). This work is presented using the ‘Strengthening the Reporting of Observational Studies in Epidemiology (STROBE)’ reporting guidelines^14^.

### Participants

#### Patient group

Persons with a diagnosis of BV, according to the Bárány Society criteria, were included^1^. Inclusion criteria were bilaterally significantly reduced or absent function of the vestibulo-ocular reflex, defined by gain values below 0.6 on the horizontal canals of the video head impulse test (vHIT), and/or the sum of the bithermal maximum peak slow phase velocity measured by caloric testing below 6°/s bilaterally, and/or rotatory chair testing with gain values smaller than 0.1 at 0.1 Hz with a maximum velocity of 50°/s. The otoliths appear to play an important role in spatial memory, and were, therefore, also assessed by means of the cervical and ocular vestibular-evoked myogenic potentials (cVEMPs and oVEMPs)^5^. However, they are not used to define the diagnosis of BV. Exclusion criteria were neurodegenerative disorders and neurodevelopmental disorders. Only persons with age-according normal hearing on at least one side were included. A minimum and maximum age of 18 and 70 years old was defined. Bessot et al. (2012) used a sample size of 12, resulting in a power of < 50%^10^. Aiming for a power of 90%, a sample size of 22 participants per group was calculated.

#### Control group

Each BV participant was matched with a healthy control subject (HC), based on age, sex, and educational level. Exclusion criteria were vestibular symptoms or dysfunctions, neurodegenerative, and neurodevelopmental disorders. Inclusion criteria were age-according normal hearing on at least one side, and horizontal vHIT gains higher than 0.8.

### 2BALANCE protocol

A detailed description of the 2BALANCE protocol was reported earlier^12^. This cognitive–motor dual-task protocol consisted of two motor tasks and seven cognitive tasks. All tasks were performed in a single-task condition (ST; without a secondary task), in a static dual-task (SDT) and a dynamic dual-task condition (DDT) combining the motor and cognitive components.

#### Motor tasks

The static motor task consisted of balancing with feet at hip-width on a force platform (Nintendo Wii Fit balance board), destabilized using a foam pad (AirEx Balance-Pad Solid). Center of pressure (CoP) data were collected using CU BrainBLoX software^15^. For each single and dual-task, the acquisition time was 30 s. However, in dual-task condition, the participants were asked to stabilize on the force platform for the entire duration of the cognitive task. The following postural parameters were analyzed using a custom-made code in MATLAB (The MathWorks, Inc., Natick, MA, USA): surface of the 95% confidence ellipse (cm^2^), the sway path length (cm), and the average velocity of the CoP displacement (cm/s). The dynamic motor task consisted of walking at a self-selected speed on a 8.8 m long GAITRite Walkway (CIR System Inc, Franklin, New Jersey). The total walking length was 11.8 m, also including 1.5 m before and 1.5 m after the walkway, in order to normalize the walking pattern. During the single and dual-task conditions, the participant walked a minimum of five lengths. The following spatiotemporal parameters were analyzed: velocity (cm/s), step length (cm), and base of support (cm).

#### Cognitive tasks

**Figure 1:**
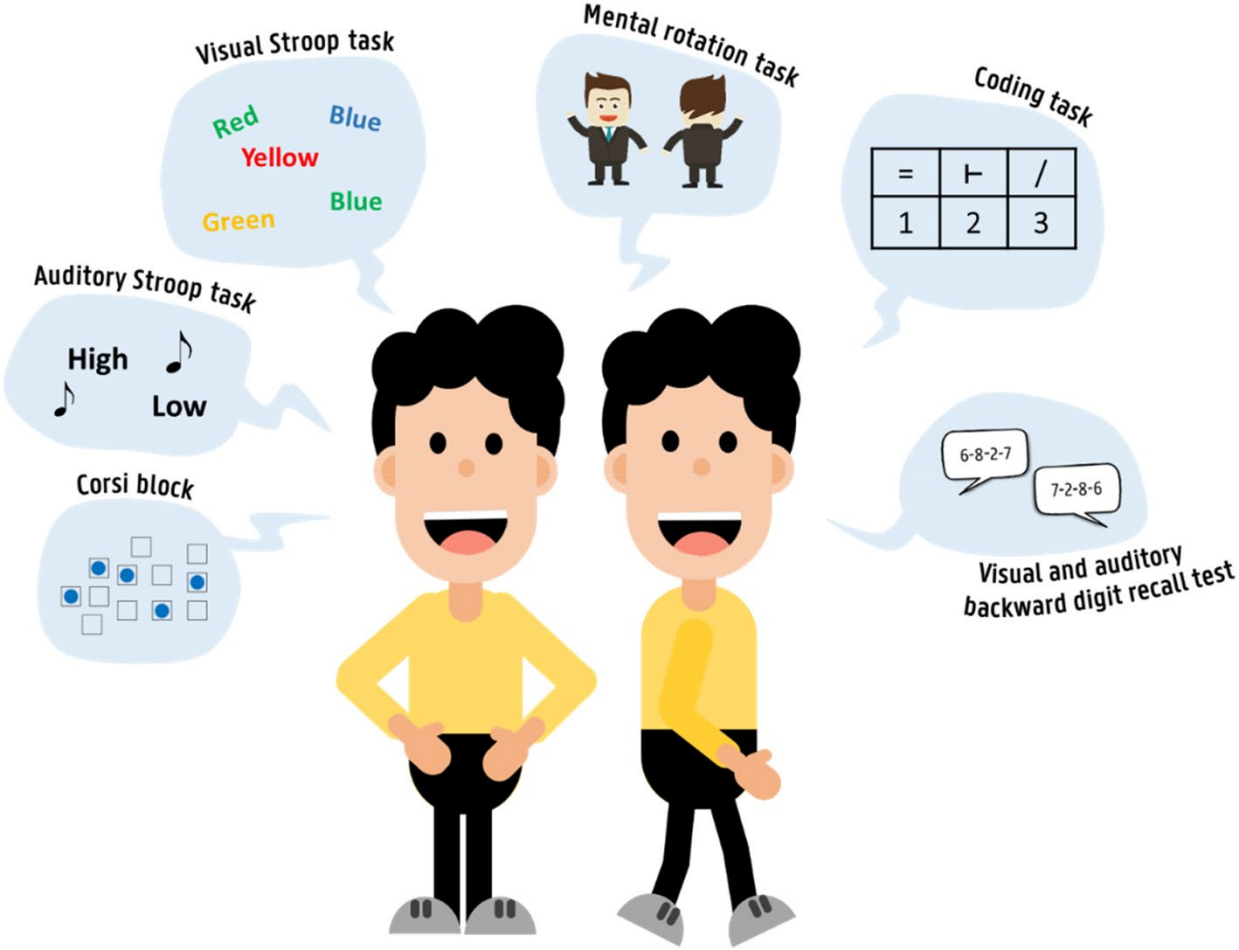
Published in Danneels et al. (2020)^7^. Visual representation of the 2BALANCE protocol, which includes the Corsi block test, the auditory and visual Stroop test, a mental rotation task, a coding task, and a visual and auditory backward digit recall test.

The following tests and cognitive domains were included in the 2BALANCE protocol (Fig. 1): the mental rotation task (percentage of incorrect responses and reaction time, mental rotation), the Corsi block test (percentage of correct responses, visuospatial memory), the coding task (number of correct responses per minute, processing speed), the visual and auditory Stroop test (reaction time, response inhibition), and the visual and auditory backward digit recall test (baseline measurement and percentage of correct responses, working memory).

### Audiovestibular assessment

The standard of care in the participating hospitals included rotatory chair testing and caloric assessment by means of electronystagmography, the vHIT, and pure-tone audiometry. At the time of participation in the 2BALANCE study, these data were acquired a year ago or less. vHIT, cVEMP, and oVEMP assessment was performed at the time of participation. Caloric assessment and rotatory chair testing were not repeated.

#### vHIT

The vHIT was performed using the ICS Impulse system (GN Otometrics, Natus). The participants focused on a visual fixation mark at one-meter distance. The examiner performed short and abrupt head thrusts in the direction of the six canals (lateral, posterior, and anterior, bilaterally). For the lateral head thrusts, the head velocity was between 150 and 250°/s, and for the vertical canals between 120 and 250°/s. Gain values were analyzed for each semicircular canal.

#### cVEMP and oVEMP

Using the Neuro-Audio system and accompanying software (Neurosoft), the cVEMP and oVEMP respectively assessed the saccule and utricle (otoliths). For the cVEMPs, air-conducted 500 Hz tone bursts with an intensity of 95 dBnHL were presented using insert earphones. For the oVEMPs, bone-conducted 500 Hz stimuli with an intensity of 140 dB force level were presented using a minishaker at Fz position (Type 4810, Brüel & Kjaer). The presence or absence of the VEMPs was noted.

#### Rotatory chair testing

The rotatory chair test assessed the lateral semicircular canals in the mid-frequencies, with 0.1 Hz and a peak velocity of 50°/s being used to interpret the gain values (Toennies Nystagliner, Ekida GmbH, ServoMed AB, and Difra Swing).

#### Caloric assessment

Bithermal caloric irrigation was performed for the stimulation of both lateral semicircular canals in the low frequencies separately (Variotherm Plus). Filtered water at 30 °C and 44 °C, was used for the cold and warm irrigations, respectively. The sum of the maximum peak slow phase eye velocity at the culmination phase (°/s) on each side was determined.

### Statistical analysis

All data were analyzed using the SPSS package 28th edition (IBM/SPSS, Chicago, IL). The student-t test and the chi-square test were used to study differences between patient and control group for continuous and dichotomous demographic data, respectively. A generalized estimating equations (GEE) model was used to investigate group differences for all cognitive and motor outcome measures. The main effects as well as the interaction effects of the group (BV and HC) and condition (ST, SDT, and DDT) were investigated. Sex, age, education level, and randomization set were included as main effects. The estimated marginal means, as well as the odds ratios (OR), and their 95% confidence intervals (CI) are presented. A negative binomial regression model was used for count data such as the number of incorrect responses on the mental rotation task and the corsi block test. For the remaining outcome variables such as reaction times and the motor outcomes, a linear model was used. The significance levels of the condition differences are presented in Tables 2 and 3, but are not discussed in detail. Significance levels below 0.05 are considered statistically significant.

## Results

### Demographic data and clinical features

Twenty-two persons with BV between the ages of 24 and 70, and twenty-two HCs with normal hearing on at least one side between the ages of 23 and 69 were included [mean age (SD), BV = 53.66 (13.35) and HC = 53.21 (13.35), 68% male]. Only on the Tinnitus Handicap Inventory (THI) and the Dizziness Handicap Inventory (DHI), a significant group difference was observed. The BV participants had a mean THI score of 13.24 (± 19.17), while the HC group had a score of 3.10 (± 9.50) (P  =  0.039). For the DHI, the BV group had a mean score of 46.60 (± 23.83), while the HC group scored 0.95 (± 3.67) (P  <  0.001). Tinnitus absence or presence, diabetes, weekly activity, noise exposure, and bilingualism were not significantly different between groups (P  >  0.05). The BV duration ranged from 0.5 to 21 years (mean  =  7.31 years). Fifty-nine percent of the BV participants met all three diagnostic criteria of BV, 32% met two, and 9% only met one criterion^1^. Additionally, cVEMPs and oVEMPs were bilaterally absent in 41% and 73%, unilaterally absent in 32% and 23%, and bilaterally present in 27% and 4%, respectively. All control subjects had horizontal vHIT gain values of  >  0.80. cVEMPs and oVEMPs were respectively bilaterally present in 95% and 91% of all control subjects, and bilaterally absent in 4.5% and 9% of all control subjects. The absent cVEMP and oVEMPs were observed in male subjects of 66 and 69 years of age. All demographic data can be found in Table 1 and the specific audiovestibular data can be found in the supplemental digital content.

**Table 1:** Demographic data and clinical features of the patient group with bilateral vestibulopathy (BV) and the healthy control group (HC).

	BV (n = 22)	HC (n = 22)	Group differences (p-values)
Age (mean)	53.66 (13.35)	53.21 (13.35)	0.911
Sex (male:female)	15:7	15:7	NA
PTA _low_right (mean)	12.98 (8.08)	9.39 (6.60)	0.120
PTA_low_left (mean)	15.78 (20.59)	10.08 (6.29)	0.222
PTA _low_best ear (mean)	10.44 (8.07)	8.18 (6.34)	0.157
BMI (mean)	25.53 (3.92)	25.93 (7.49)	0.833
Diabetes	0 (0%)	1 (5%)	0.312
Weekly physical activity (hours)	5.81	6.24	0.887
Noise exposure	6 (27%)	4 (18%)	0.719
Bilingualism	3 (14%)	1 (5%)	0.345
Tinnitus presence	10 (45%)	5 (23%)	0.197
Tinnitus handicap inventory	13.24 (19.17)	3.10 (9.50)	**0.039**
Dizziness handicap inventory	46.60 (23.83)	0.95 (3.67)	** < 0.001**
BV etiologies (%)			
Idiopathic	18 (82%)	NA	NA
Auto-immune	2 (9%)	NA	NA
Ototoxicity	1 (5%)	NA	NA
Suspicion of bilateral vestibular neuritis	1 (5%)	NA	NA
Specifications degree of BV			
All three Bárány criteria met	13 (59%)	0 (0%)	NA
Two Bárány criteria met	7 (32%)	0 (0%)	NA
One Bárány criterion met	2 (9%)	0 (0%)	NA

The mean values and standard deviations are presented for all data. Significant p-values are indicated in bold.

### Cognitive tasks

**Figure 2:**
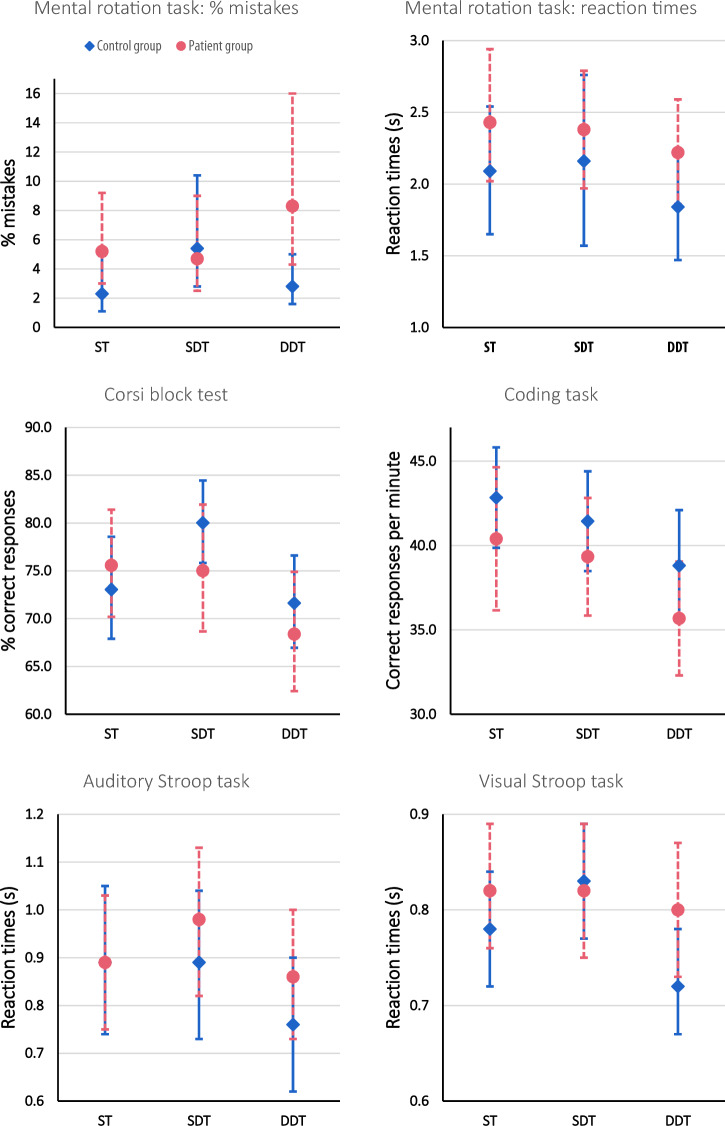
The estimated marginal means and their error bars for the cognitive data. The pink error bars present the patient data, while the blue error bars present the healthy control data. These data are presented for each cognitive task in single-task (ST), static dual-task (SDT), and dynamic dual-task (DDT) condition.

Descriptive data and error bars for the cognitive tasks can be found in Table 2 and Fig. 2.

**Table 2:** Descriptive data for the cognitive tasks.

	ST	SDT	DDT	Condition differences (p-values)	Interaction (p-values)
Mental rotation task (percentage of mistakes, %)					
BV	5.24 [2.98–9.20]	4.72 [2.49–8.95]	8.26 [4.25–16.03]	ST & SDT = 0.752 ST & DDT = 0.100 SDT & DDT = 0 .052	ST → SDT = **0.023** ST → DDT = 0.620 SDT → DDT = **0.012**
HC	2.28 [1.06–4.9]	5.36 [2.75–10.43]	2.79 [1.57–4.95]	ST & SDT = **0.001** ST & DDT = 0.651 SDT & DDT = 0.099	
Group differences (p-value)	**0.024**	0.730	** < 0.001**		
Mental rotation task (response time, s)					
BV	2.43 [2.02–2.94]	2.38 [1.97–2.79]	2.22 [1.86–2.59]	ST & SDT = 0 .521 ST & DDT = **0.007** SDT & DDT = **0.014**	ST → SDT = 0.319 ST → DDT = 0.708 SDT → DDT = 0 .328
HC	2.09 [1.65–2.54]	2.16 [1.57–2.76]	1.84 [1.47–2.21]	ST & SDT = 0.444 ST & DDT = **0.021** SDT & DDT =**0** **.039**	
Group differences (p-value)	0.252	0.536	0.093		
Corsi block (percentage of correct responses, %)					
BV	75.58 [70.19–81.40]	75.01 [68.67–81.93]	68.38 [62.42–74.90]	ST & SDT = 0.754 ST & DDT = **0.010** SDT & DDT = **0** **.022**	ST → SDT = **0.017** ST → DDT = 0.095 SDT → DDT = 0 .712
HC	73.04 [67.90–78.56]	80.02 [75.83–84.45]	71.62 [66.96–76.61]	ST & SDT = **0.007** ST & DDT = 0.495 SDT & DDT = ** < 0.001**	
Group differences (p-value)	0.427	0.133	.328		
Coding task (responses per minute)					
BV	40.40 [36.16–44.64]	39.34 [38.85–42.82]	35.68 [32.30–39.05]	ST & SDT = 0.188 ST & DDT = ** < 0.001** SDT & DDT = ** < 0.001**	ST → SDT = 0.761 ST → DDT = 0.574 SDT → DDT =0 .420
HC	42.84 [39.86–45.82]	41.44 [38.49–44.40]	38.81 [35.53–42.10]	ST & SDT = 0.066 ST & DDT = ** < 0.001** SDT & DDT = ** < 0.001**	
Group differences (p-value)	0.195	0.219	0.078		
Auditory Stroop task (response time, s)					
BV	0.89 [0.75–1.03]	0.98 [0.82–1.13]	0.86 [0.73–1.00]	ST & SDT = **0.001** ST & DDT = 0.432 SDT & DDT = ** < 0.001**	ST → SDT = **0.023** ST → DDT = **0.015** SDT → DDT = 0 .750
HC	0.89 [0.74–1.05]	0.89 [0.73–1.04]	0.76 [0.62–0.90]	ST & SDT = 0.822 ST & DDT = ** < 0.001** SDT & DDT = ** < 0.001**	
Group differences (p-value)	0.907	0.300	0.097		
Visual Stroop task (response time, s)					
BV	0.82 [0.76–0.89]	0.82 [0.75–0.89]	0.80 [0.73–0.87]	ST & SDT = 0.857 ST & DDT = 0.326 SDT & DDT = 0.520	ST → SDT = 0 .204 ST → DDT = 0.330 SDT → DDT = **0.027**
HC	0.78 [0.72–0.84]	0.83 [0.77–0.89]	0.72 [0.67–0.78]	ST & SDT = **0.005** ST & DDT = **0.005** SDT & DDT = ** < 0 .001**	
Group differences (p-value)	0.234	0.826	**0.032**		

The estimated marginal means and 95% confidence interval [CI] are presented. Additionally, the p-values for condition differences and group differences are presented. All significant p-values are indicated in bold.

#### Mental rotation task (percentage of mistakes and reaction times)

Patients made more mistakes on the mental rotation task in ST and DDT setting than their matched controls. The estimated mean mistake rate was 2.30 and 2.96 times higher in the patient group compared to the control group for the ST and DDT, respectively (95% CI [1.12–4.73], P  =  0.024 and 95% CI [1.57–5.59], P  <  0.001; respectively). For the patient group, the mistake rate decreased by 0.52% between ST and SDT, while an increase of 3.1% was observed for the control group (interaction: P  =  0.023). Comparing SDT with DDT, the percentage of mistakes increased by 3.6% in the BV group, while a decrease of 2.6% was noted for the HC group (P  =  0.012). Despite the BV group having slower reaction times than the HC group in all different conditions, no significant group difference nor any interaction effect was noted.

#### Corsi block

For the Corsi block, no group differences were found in any condition. An interaction effect was noted between the ST and SDT condition; the number of correct responses decreased by 0.57% in the patient group, but increased by 6.98% in the control group (P  =  0.017).

#### Coding task

Even though the BV group had a systematically lower number of correct responses per minute in each condition, no significant group differences, nor any interaction effects were observed.

#### Auditory Stroop task

Even though no group differences were observed, significant interaction effects were found. The reaction times of the patient group increased by 0.09 s between ST and SDT, while those of the control group were identical (P  =  0.023). Additionally, an interaction effect was noted between ST and DDT, where the reaction times decreased by 0.03 s for the patient group and decreased by 0.13 s for the control group (interaction: P  =  0.015).

#### Visual Stroop task

A group difference was only observed for the DDT condition (95%CI [1.01–1.16], P  =  0.032), for which the reaction times of the BV group were 1.08 times higher than for the HC group. A significant interaction effect was observed between SDT and DDT condition. The reaction time decreased by 0.02 s for the BV group and by 0.11 s for the HC group (interaction: P  =  0.027).

#### Auditory and visual backward digit recall test

A baseline measurement was first administered for the auditory and visual version separately, to establish the length for which at least 3 out of 5 items were repeated correctly in reverse order, while seated. For the auditory version, the means and standard deviations for the BV and HC group were respectively 4.50 (SD  =  1.10) and 5.09 (SD  =  0.87). For the visual version, the means and standard deviations for the BV and HC group were respectively 4.45 (SD  =  1.22) and 5.41 (SD  =  0.96). Using a student-t test, statistically significant group differences were observed (P  =  0.028 and P  =  0.003, respectively). Group differences could not be calculated for the SDT and DDT condition given the different baseline measurements. No significant interaction effects were observed.

### Motor tasks

**Figure 3:**
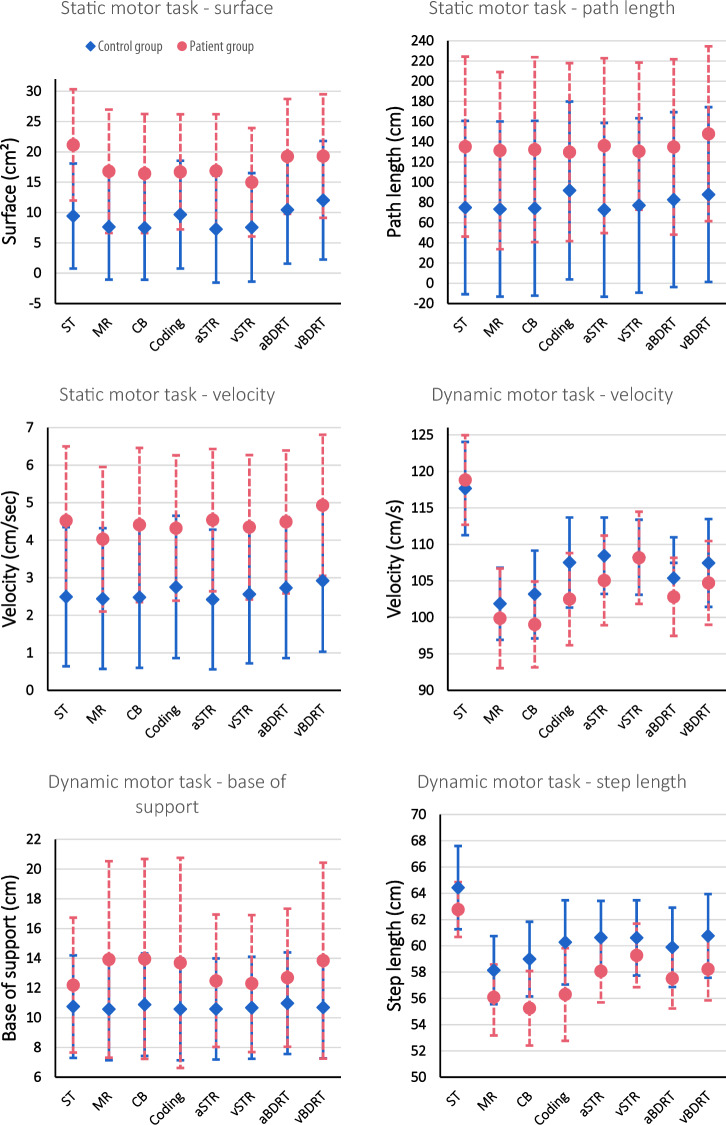
The estimated marginal means and their error bars for the motor data. The pink error bars present the patient data, while the blue error bars present the healthy control data. The static and dynamic motor data are presented for the single-task (ST) condition and while dual-tasking combined with the different cognitive tasks: the mental rotation task (MR), the Corsi block (CB), the coding task, the auditory and visual Stroop task (aSTR and vSTR), and the auditory and visual backward digit recall test (aBDRT and vBDRT).

Descriptive data and error bars for the motor tasks can be found in Table 3 and Fig. 3.

**Table 3:** Descriptive data for the static and dynamic motor tasks during the single task (ST) and dual-tasks (DT) are presented.

	Static motor data (Wii balance board)							
	ST	SDT_MR	SDT_CB	SDT_aSTR	SDT_vSTR	SDT_Coding	SDT_aBDRT	SDT_vBDRT
Surface (cm^2^)								
BV	21.15 [11.98–30.33]	16.79 [6.61–26.96]	16.42 [6.60–26.25]	16.86 [7.50–26.21]	14.98 [6.04–23.92]	16.70 [7.22–26.19]	19.25 [9.78–28.72]	19.30 [9.12–29.49]
HC	9.42 [0.77–18.08]	7.62 [− 1.06–16.31]	7.48 [− 1.08–16.03]	7.25 [− 1.56–16.06]	7.55 [− 1.40–16.51]	9.65 [0.76–18.54]	10.44 [1.57–19.31]	12.02 [2.25–21.79]
Group differences (p-value)	** < 0.001**	** < 0.001**	** < 0.001**	** < 0.001**	** < 0.001**	**0.012**	**0.003**	**0.026**
Condition difference compared to ST (BV)	NA	**0.047**	**0.043**	0.086	**0.016**	0.071	0.440	0.509
Condition difference compared to ST (HC)	NA	0.117	0.103	**0.010**	0.229	0.882	0.450	0.111
Path length (cm)								
BV	135.26 [46.21–224.30]	131.40 [33.70–209.09]	132.25 [40.76–223.74]	136.21 [49.63–222.78]	130.58 [72.74–218.41]	129.82 [41.75–217.88]	134.93 [48.08–221.78]	147.97 [61.47–234.47]
HC	74.96 [− 10.81–160.74]	73.43 [− 13.19–160.05]	74.21 [− 12.28–160.70]	72.72 [− 13.30–158.74]	77.05 [− 9.24–163.34]	91.82 [3.81–179.83]	82.69 [− 3.97–169.35]	87.81 [1.35–174.27]
Group differences (p-value)	** < 0.001**	** < 0.001**	** < 0.001**	** < 0.001**	** < 0.001**	** < 0.001**	** < 0.001**	** < 0.001**
Condition difference compared to ST (BV)	NA	0.124	0.779	0.943	0.538	0.646	0.972	0.269
Condition difference compared to ST (HC)	NA	0.665	0.821	0.488	0.674	**0.025**	0.102	**0.047**
Velocity (cm/s)								
BV	4.52 [2.53–6.50]	4.03 [2.10–5.95]	4.41 [2.35–6.46]	4.54 [2.64–6.43]	4.35 [2.42–6.27]	4.32 [2.39–6.26]	4.49 [2.58–6.39]	4.93 [3.05–6.81]
HC	2.49 [0.64–4.35]	2.44 [0.57–4.32]	2.48 [0.60–4.35]	2.42 [0.56–4.28]	2.56 [0.72–4.41]	2.75 [0.86–4.65]	2.73 [0.86–4.61]	2.92 [1.03–4.82]
Group differences (p-value)	** < 0.001**	** < 0.001**	** < 0.001**	** < 0.001**	** < 0.001**	** < 0.001**	** < 0.001**	** < 0.001**
Condition difference compared to ST (BV)	NA	0.105	0.759	0.961	0.516	0.629	0.924	0.281
Condition difference compared to ST (HC)	NA	0.677	0.899	0.513	0.657	0.118	0.072	**0.044**

	Dynamic motor data (GAITRite Walkway)							
	ST	DDT_MR	DDT_CB	DDT_aSTR	DDT_vSTR	DDT_Coding	DDT_aBDRT	DDT_vBDRT
Velocity (cm/s)								
BV	118.82 [112.69–124.96]	99.87 [93.04–106.69]	99.03 [93.15–104.91]	105.06 [98.92–1111.20]	108.17 [101.85–114.48]	102.50 [96.18–108.82]	102.81 [97.46–108.16]	104.72 [98.99–110.45]
HC	117.66 [111.27–124.04]	101.87 [96.92–106.81]	103.18 [97.21–109.15]	108.45 [103.21–113.68]	108.24 [103.10–113.38]	107.51 [101.34–113.69]	105.37 [99.77–110.97]	107.46 [101.44–113.48]
Group differences (p-value)	0.765	0.581	0.234	0.353	0.982	0.178	0.447	0.450
Condition difference compared to ST (BV & HC)	NA	** < 0.001**	** < 0.001**	** < 0.001**	** < 0.001**	** < 0.001**	** < 0.001**	** < 0.001**
Step length (cm)								
BV	62.76 [60.67–64.86]	56.08 [53.18–58.58]	55.25 [52.41–58.08]	58.07 [55.69–60.45]	59.27 [56.85–61.69]	56.30 [52.77–59.82]	57.51 [55.23–59.79]	58.22 [55.85–60.59]
HC	64.43 [61.26–67.60]	58.14 [55.55–60.74]	58.99 [56.15–61.83]	60.63 [57.83–63.42]	60.61 [57.74–63.47]	60.27 [57.06–63.47]	59.89 [56.87–62.91]	60.76 [57.57–63.94]
Group differences (p-value)	0.247	0.122	**0.020**	0.074	0.349	**0.037**	0.101	0.103
Condition difference compared to ST (BV & HC)	NA	** < 0.001**	** < 0.001**	** < 0.001**	** < 0.001**	** < 0.001**	** < 0.001**	** < 0.001**
Base of support (cm)								
BV	12.19 [7.66–16.73]	13.92 [7.31–20.53]	13.95 [7.23–20.68]	12.48 [8.03–16.94]	12.30 [7.69–16.91]	13.69 [6.62–20.76]	12.70 [8.05–17.34]	13.84 [7.25–20.43]
HC	10.75 [7.30–14.20]	10.57 [7.14–14.00]	10.88 [7.43–14.33]	10.59 [7.19–13.99]	10.67 [7.24–14.10]	10.58 [7.14–14.00]	10.97 [7.56–14.39]	10.69 [7.27–14.11]
Group differences (p-value)	0.141	0.106	0.149	**0.049**	0.103	0.173	0.089	0.128
Condition difference compared to ST (BV)	NA	0.180	0.191	0.282	0.687	0.323	0.041	0.200
Condition difference compared to ST (HC)	NA	0.370	0.535	0.390	0.691	0.387	0.357	0.763

The following cognitive tasks were performed during the static dual-task (SDT) and the dynamic dual-task (DDT): the mental rotation task (MR), the Corsi block (CB), the auditory and visual Stroop task (aSTR and vSTR), the coding task, and the auditory and visual backward digit recall test (aBDRT and vBDRT). The estimated marginal means and 95% confidence interval [CI] are presented. Additionally, the p-values for condition differences and group differences are presented. All significant p-values are indicated in bold.

#### Static dual-task

Group differences were observed in the ST as well as all DT conditions for all outcome parameters (surface, path length, and velocity). All parameters were consistently significantly higher in the BV group compared to the HC group (P  <  0.001 to 0.026). Additionally, condition differences were observed in both groups, where a larger surface and/or longer path length were noted in ST condition compared to several DT conditions (P  =  0.010 to 0.047). No significant interaction effects were observed.

#### Dynamic dual-task

Even though a tendency for slower gait, smaller step length, and a larger base of support was observed for the BV group throughout all conditions, no significant group differences were noted, with the exception of step length during the Corsi block and during the coding task (P  =  0.020 and P  =  0.037, respectively), and the base of support during the auditory Stroop task (P  =  0.049). A smaller step length and wider base of support were observed in the BV group. For both groups, the velocity and step length were consistently higher for the ST compared to all DTs (Table 3). No significant interaction effects were observed.

## Discussion

Cognitive–motor dual-tasking was evaluated prospectively in a BV population and compared with a healthy control group matched for age, sex, hearing status, and educational level. Poorer mental rotation skills and working memory were observed in single-task condition in the BV group. Additionally, poorer mental rotation skills and visual response inhibition were observed in dynamic dual-task condition. Increased cognitive–motor interference, resulting in a decrease in cognitive performance in the BV group, was observed for mental rotation, response inhibition, and auditory working memory. For the static motor task, the BV group used a bigger surface, longer path length, and higher velocity to remain balanced throughout all single and dual-task conditions. No group differences were noted for the dynamic motor task.

According to *Kahneman’s Capacity Model of Attention*, dual-task studies enable observing the allocation of attention to cognitive and motor tasks^9^. One of the theories proposed to explain the neurophysiological mechanisms behind cognitive–motor interference is the *capacity sharing theory*^16^. This theory states that the performance of the motor and/or cognitive task will decrease when the available cognitive capacity is surpassed. This decrease in performance depends on prioritization. In order to observe the spontaneous allocation of attention, no instructions were given about prioritization in this study. A larger cognitive–motor interference was observed in the BV group, resulting in a decrease in cognitive performance. No interaction effects were observed for the motor tasks, which could be in line with the *posture first strategy*. This might indicate that the BV group prioritized the postural task, in order to minimize danger^17^.

### Cognitive performance

Out of all cognitive domains, visuospatial cognition has been studied most frequently in persons with BV. This domain can be subdivided into visuospatial memory, mental rotation, and visuospatial navigation. Brandt et al. (2005) observed a significant hippocampal decrease in acquired BV compared to healthy controls, associated with spatial memory and navigation issues. The current study was unable to identify a significant group difference on the *visuospatial memory* task. These findings are in agreement with the Corsi block test performed by Popp et al. (2017), where patients with BV did not score significantly different from the control group^8^. In contrast, a more recent study by Ahmad et al. (2022) did observe lower scores on the Corsi block test in persons with BV, compared to HCs^18^. In the current study, adding a walking task elicited a significantly lower score in the patient group, but not in the control group. This might indicate that the walking task required a certain amount of cognitive capacity, causing the remaining capacity to be too little to perform the cognitive task in the same manner as while seated. Similar to the results reported by Grabherr et al. (2011), impairment in the second visuospatial subdomain, *mental rotation*, was observed^19^. Additionally, similar to the visuospatial memory task, adding a walking task elicited a significantly higher percentage of incorrect responses compared to the SDT. However, this interaction effect might have been elicited by the remarkably high number of mistakes in SDT in the control group. The third visuospatial subdomain; *visuospatial navigation*, was not included in the 2BALANCE protocol as tasks assessing this domain, such as the virtual Morris water maze task require a manual response, and would have influenced motor performance while dual-tasking^20^.

Aside from the visuospatial domains, this study also assessed working memory, response inhibition, and processing speed. The current study observed impairment in the *visual and auditory working memory* domain in ST condition. Auditory working memory had only been assessed by Ahmad et al. (2022)^18^. However, no significant differences were found between BV and HC group. Bosmans et al. (2022) did observe deficits on the immediate memory subscale of the Repeatable Battery for the Assessment of Neuropsychological Status for Hearing-Impaired Individuals (RBANS-H) in an older adult population with BV, but did not observe deficits in the delayed memory subscale^21^. Assessing the cognitive subdomain *response inhibition*, Popp et al. (2017) observed impairment on the visual Stroop task^8^. This is in contrast with the current results and the study by Ahmad et al. (2022), where the Stroop tasks did not elicit any group differences. However, the study by Popp et al. (2017) lacked information on hearing status, while the other two studies only included persons with BV and normal hearing. Therefore, the possible contribution of hearing loss in the study by Popp et al. (2017) cannot be ruled out. The significant interaction effects on the Stroop tasks in the current study are in line with the visuospatial tasks, where adding a motor task elicited a difference in cognitive performance between both groups. Specifically, the control group responded significantly faster while walking, while there was no observed difference between both conditions for the patient group. These faster response times in the control group might have resulted from a self-imposed time limit to finish the tasks before reaching the end of the GAITRite walkway. However, the patient group might have been less able to meet this time limit, because of limited cognitive and motor resources. Finally, no significant observations could be noted for the cognitive subdomain *processing speed*. This contrasts with Popp et al. (2017), where poorer processing speed was noted for the BV group, using the theory of visual attention.

It could be suspected that the BV group might experience difficulties with visually stabilizing the cognitive stimuli while walking, which could influence their cognitive performance. However, cognitive–motor interference was observed in the visual as well as auditory tasks. Additionally, the coding task required adequate visual scanning, and might, therefore, be most susceptible to oscillopsia. However, no significant differences were identified for this task. Based on these data, it appears to be unlikely that the cognitive deficits are solely caused by the presence of oscillopsia.

### Motor performance

Similar to the selection of the different cognitive tasks, this study used two different postural tasks. During the static postural task, the BV group used a bigger surface, longer path length, and higher velocity to remain balanced during the static motor task in all test conditions. It could be hypothesized that postural control would decrease with an added cognitive task. However, especially for the outcome parameter *surface*, a converse effect could be noted. The constrained-action hypothesis could explain this phenomenon, which is observed in both groups. When asked to balance as steadily as possible, internal attentional focus on this task could negatively affect the postural balance. When a cognitive task is simultaneously performed, the attention might be drawn away from the balancing task and might restore the postural automaticity^22,23^. Stoffregen et al. (1999) proposed that the presence of a visual fixation mark may help to regulate balance^24^. However, the lack of difference in the CoP displacement between the visual and auditory tasks does not support this theory in the current study.

In the dynamic dual-task condition, participants were asked to walk at a self-selected and comfortable walking speed. This might explain the similarity of the gait velocity of both groups. A preferred walking speed similar to healthy persons has previously been reported, as the vestibular influence on lower limb muscles might be selectively suppressed by increasing the walking speed^25^. Therefore, maintaining a slower gait-speed might be more challenging in the BV population and might elicit a difference in gait pattern.

### Limitations and future perspectives

This study aimed to assess the impact of an isolated vestibular dysfunction on cognitive and motor performance. Therefore, persons with hearing loss were excluded from participating. Given the high comorbidity between vestibular loss and hearing loss, studying their combined impact on cognitive–motor performance would be an added value^26,27^. A first limitation of this study was the significant group difference on the THI, with a higher score for the patient compared to the control group. It is known that tinnitus can also influence cognitive performance. Specifically, listening effort is higher in normal-hearing persons with tinnitus compared to normal-hearing persons without tinnitus^28^. However, this could only have had an impact on the auditory tasks, which we were unable to identify. Second, this study aimed to implement a comprehensive protocol, assessing several different cognitive domains. However, social cognition and language were not included in the 2BALANCE protocol. Future research involving these domains could create a more complete cognitive picture of persons with BV. Third, although the sample size calculation estimated the number of participants to be sufficient, a larger sample size might elicit more group differences, where currently only tendencies were observed. Finally, all participants underwent vestibular assessment of the semicircular canals and the otolith organs. In spite of the growing evidence for the importance of the otolith organs in cognition, it is not within the scope nor the possibility of the current research to unravel the contribution of these organs on cognitive and motor performance.

### Supplementary Information

Supplementary Information.

## Data Availability

The authors confirm that the necessary data supporting the findings of this study are available within the article. The raw data files of this study are available from the corresponding author, MD, upon reasonable request. The audiofiles for the cognitive tests are not available due to ethical reasons.
