# Supplementary material for: The impact of vestibular function on cognitive–motor interference: a case–control study on dual-tasking in persons with bilateral vestibulopathy and normal hearing
Source: Sci Rep. 2023 Aug 23;13:13772. doi: 10.1038/s41598-023-40465-2 (PMC10447548; doi:10.1038/s41598-023-40465-2)
Supplement: Supplementary file 1 — Supplementary Information. [file 41598_2023_40465_MOESM1_ESM.docx]

**Audiovestibular data.** For the torsion swing, the caloric assessment, and the video Head Impulse Test (vHIT), the results are indicated in red when complying with the Bárány Society Diagnostic criteria. Specifically, rotatory chair gain values are below 0.1 (torsion swing test at 0.1 Hz, Vmax = 50°/sec), the sum of the maximal peak slow phase eye velocity of the caloric assessment is below 6°/s, and vHIT gain values of the lateral canals are below 0.6 when indicated in red. Results above these cut-off values are indicated in green. Absent cervical and ocular vestibular-evoked myogenic potentials (cVEMPs and oVEMPs) are indicated in red, and present and reproducible VEMPs are indicated in green.

|  | **Etiology** | **PTA_low_ best ear** | **PTA_high_**  **best ear** | **Grade**  **hearing loss** | **Torsion swing** | **Sum caloric right** | **Sum caloric left** | **vHIT lateral right** | **vHIT lateral left** | **cVEMP right** | **cVEMP left** | **oVEMP right** | **oVEMP left** |
| --- | --- | --- | --- | --- | --- | --- | --- | --- | --- | --- | --- | --- | --- |
| **Bilateral vestibulopathy and normal hearing** | | | | | | | | | | | | | |
| BV_01 | Idiopathic | 6.66 | 8.33 | Age-according |  |  |  |  |  |  |  |  |  |
| BV_02 | Idiopathic | 3.33 | 20 | Age-according |  |  |  |  |  |  |  |  |  |
| BV_03 | Idiopathic | 10 | 11.67 | Age-according |  |  |  |  |  |  |  |  |  |
| BV_04 | Idiopathic | 1.67 | 5 | Age-according |  |  |  |  |  |  |  |  |  |
| BV_05 | Idiopathic | 3.33 | 8.33 | Age-according |  |  |  |  |  |  |  |  |  |
| BV_06 | Idiopathic | 16.67 | 18.33 | Age-according |  |  |  |  |  |  |  |  |  |
| BV_07 | Idiopathic | 6.67 | 6.67 | Age-according |  |  |  |  |  |  |  |  |  |
| BV_08 | Idiopathic | 10 | 25 | Age-according |  |  |  |  |  |  |  |  |  |
| BV_09 | Suspicion bilateral vestibular neuritis | 6.67 | 11.67 | Age-according |  |  |  |  |  |  |  |  |  |
| BV_10 | Idiopathic | 18.33 | 26.67 | Age-according |  |  |  |  |  |  |  |  |  |
| BV_11 | Idiopathic | 21.67 | 26.67 | Age-according |  |  |  |  |  |  |  |  |  |
| BV_12 | Idiopathic | 1.33 | 0 | Age-according |  |  |  |  |  |  |  |  |  |
| BV_13 | Idiopathic | 36.67 | 30 | Age-according |  |  |  |  |  |  |  |  |  |
| BV_14 | Idiopathic | 13.33 | 21.67 | Age-according |  |  |  |  |  |  |  |  |  |
| BV_15 | Idiopathic | 16.67 | 18.33 | Age-according |  |  |  |  |  |  |  |  |  |
| BV_16 | Idiopathic | 5 | 8.33 | Age-according |  |  |  |  |  |  |  |  |  |
| BV_17 | Idiopathic | 8 | 10 | Age-according |  |  |  |  |  | N/A | N/A |  |  |
| BV_18 | Ototoxicity | 6.67 | 16.67 | Age-according |  |  |  |  |  |  |  |  |  |
| BV_19 | Idiopathic | <20 | <20 | Age-according |  |  |  |  |  |  |  |  |  |
| BV_20 | Idiopathic | 10 | 18 | Age-according |  |  |  |  |  |  |  |  |  |
| BV_21 | Auto-immune | 5 | 15 | Age-according |  |  |  |  |  |  |  |  |  |
| BV_22 | Auto-immune | 5 | 5 | Age-according |  |  |  |  |  |  |  |  |  |
| **Healthy control subjects** | | | | | | | | | | | | | |
| HC_01 | N/A | 10 | 8.33 | Age-according | N/A | N/A | N/A |  |  |  |  |  |  |
| HC_02 | N/A | 18.33 | 26.67 | Age-according | N/A | N/A | N/A |  |  |  |  |  |  |
| HC_03 | N/A | 5 | 10 | Age-according | N/A | N/A | N/A |  |  |  |  |  |  |
| HC_04 | N/A | 6.67 | 8.33 | Age-according | N/A | N/A | N/A |  |  |  |  |  |  |
| HC_05 | N/A | 15 | 25 | Age-according | N/A | N/A | N/A |  |  |  |  |  |  |
| HC_06 | N/A | 3.33 | 5 | Age-according | N/A | N/A | N/A |  |  |  |  |  |  |
| HC_07 | N/A | 10 | 10 | Age-according | N/A | N/A | N/A |  |  |  |  |  |  |
| HC_08 | N/A | 5 | 8.33 | Age-according | N/A | N/A | N/A |  |  |  |  |  |  |
| HC_09 | N/A | 5 | 0 | Age-according | N/A | N/A | N/A |  |  |  |  |  |  |
| HC_10 | N/A | 0 | -3.33 | Age-according | N/A | N/A | N/A |  |  |  |  |  |  |
| HC_11 | N/A | 18.33 | 16.67 | Age-according | N/A | N/A | N/A |  |  |  |  |  |  |
| HC_12 | N/A | 0 | 1.67 | Age-according | N/A | N/A | N/A |  |  |  |  |  |  |
| HC_13 | N/A | 8.33 | 13.33 | Age-according | N/A | N/A | N/A |  |  |  |  |  |  |
| HC_14 | N/A | -3.33 | -1.67 | Age-according | N/A | N/A | N/A |  |  |  |  |  |  |
| HC_15 | N/A | 13.33 | 16.67 | Age-according | N/A | N/A | N/A |  |  |  |  |  |  |
| HC_16 | N/A | 16.67 | 11.66 | Age-according | N/A | N/A | N/A |  |  |  |  |  |  |
| HC_17 | N/A | 10 | 15 | Age-according | N/A | N/A | N/A |  |  |  |  |  |  |
| HC_18 | N/A | 1.67 | 1.67 | Age-according | N/A | N/A | N/A |  |  |  |  |  |  |
| HC_19 | N/A | 11.67 | 16.66 | Age-according | N/A | N/A | N/A |  |  |  |  |  |  |
| HC_20 | N/A | 13.33 | 26.67 | Age-according | N/A | N/A | N/A |  |  |  |  |  |  |
| HC_21 | N/A | 11.67 | 11.67 | Age-according | N/A | N/A | N/A |  |  |  |  |  |  |
| HC_22 | N/A | 0 | 5 | Age-according | N/A | N/A | N/A |  |  |  |  | N/A | N/A |

*Abbreviations: Not applicable (N/A), Pure Tone Average (PTA), video Head Impulse Test (vHIT),* *cervical and ocular vestibular-evoked myogenic potentials (cVEMPs and oVEMPs*), *bilateral vestibulopathy (BV), hearing loss (HL), normal-hearing (NH), and healthy control (HC).*
